# Context or composition: How does neighbourhood deprivation impact upon adolescent smoking behaviour?

**DOI:** 10.1371/journal.pone.0192566

**Published:** 2018-02-08

**Authors:** Tim Morris, David Manley, Maarten Van Ham

**Affiliations:** 1 MRC Integrative Epidemiology Unit, University of Bristol, Bristol, United Kingdom; 2 School of Geographical Sciences, University of Bristol, Bristol, United Kingdom; 3 OTB—Research for the Built Environment, Faculty of Architecture and the Built Environment, Delft University of Technology, Delft, The Netherlands; 4 School of Geography and Sustainable Development, University of St Andrews, Fife, United Kingdom; CUNY, UNITED STATES

## Abstract

Neighbourhood effects studies have demonstrated an association between area deprivation and smoking behaviour whereby people living in deprived neighbourhoods are more likely to smoke than those in non-deprived neighbourhoods. This evidence though is based largely upon data that ignores long term exposures to neighbourhood contexts and is confounded by neighbourhood selection bias. In this study, we investigate the temporal ordering of exposure to neighbourhood deprivation throughout childhood and whether associations between neighbourhood deprivation and cigarette smoking are due to compositional or contextual neighbourhood effects. Data come from a UK cohort study, the Avon Longitudinal Study of Parents and Children (ALSPAC). We use longitudinal measures of neighbourhood deprivation and self-reported smoking behaviour for 2744 children to examine the influence of neighbourhood deprivation on smoking status and smoking heaviness at age 17. Our results demonstrate that children who are born into and grow up in deprived neighbourhoods are up to twice as likely to be smokers at age 17 than those in non-deprived neighbourhoods. These associations are largely due to family socioeconomic position and the intergenerational transmission of smoking behaviour from parents to children; compositional rather than direct contextual ‘neighbourhood effects’. Our findings highlight the importance of considering longitudinal exposure to neighbourhood deprivation over cross sectional exposure. In conclusion, we find that it is the family rather than the neighbourhood into which a child is born that determines their smoking behaviour.

## Introduction

Over the past two decades there has been an increase in research examining how neighbourhoods impact the health of their resident individuals. There is now a large neighbourhood effects literature which suggests that living in deprived neighbourhoods or those characterised by low socioeconomic status is associated with poor health outcomes [1,2]. One domain of health that has received particular focus is that of health damaging behaviours, such as cigarette smoking, which are robustly associated with poor health. Health damaging behaviours have provided fertile ground for research because they are modifiable–interventions can lead to behaviour adaptation–and therefore offer a source of preventable morbidity and mortality [3]. The neighbourhood effects literature has revealed that people who live in more deprived neighbourhoods are more likely to smoke and less likely to quit than those who live in less deprived neighbourhoods [4–6]. Because neighbourhood factors are malleable to policy interventions, they present a strong opportunity for social change that can have downstream health consequences for populations.

The relationship between neighbourhood deprivation and smoking behaviour is complex, with multiple mechanisms and social pathways through which neighbourhood effects may operate. General neighbourhood effects mechanisms are summarised by Galster under four categories; social-interactive, environmental, geographical and institutional [7]. Social-interactive mechanisms refer to the ways in which the social environment and interactions within this can influence individual behaviour, and may include social norms and psychosocial stress [8]; neighbourhood peer behaviour [9]; and the embeddedness of neighbourhood smoking behaviours [10]. Environmental mechanisms refer to the manmade and natural place-based characteristics of a neighbourhood, and can influence smoking through characteristics such as neighbourhood poverty, crime, and disorder [11,12]. Geographical and institutional mechanisms, which refer to spatial elements and the role played by institutions or organisational entities respectively, have focussed largely on the location and density of tobacco retailers. Tobacco retailers are more spatially concentrated in deprived than non-deprived neighbourhoods, leading to greater access to cigarettes in these areas which may lead to higher smoking rates [13]. These mechanisms are not exhaustive nor necessarily distinct from each other; overlap or interaction may exist between them.

While theory has advanced to offer explanations for the association between neighbourhood deprivation and smoking behaviour, methodological and empirical advances have been slower [14]. The evidence for neighbourhood effects on smoking is based largely upon cross sectional and short time span longitudinal data measuring limited duration of exposure. As a result, inferences drawn from this data are associational and unsuitable for identifying directional (casual) neighbourhood effects. By attributing exposure at just one occasion, or for a short period, these studies provide only a snapshot of associations that ignores longer term exposures to neighbourhood contexts. Consequently, they fail to capture any information on change in exposures, and are subject to temporal confounding in addition to more classical forms of confounding bias. This is a critical limitation given that exposure to neighbourhood deprivation can change throughout the life course, either through residential mobility leading to the experience of multiple neighbourhoods, or the neighbourhood around the individual changing. Recent work has highlighted this problem by demonstrating that cross sectional neighbourhood factors paint an overly simplistic and unrealistic picture of longer term neighbourhood exposure [15].

Cohort studies, with longer term longitudinal follow up are explicitly able to explore longer periods of exposure and are protected from temporal confounding. Longitudinal studies into the impact of neighbourhood deprivation on health behaviours have revealed conflicting findings [16–18], mirroring those on the broader social patterning of health behaviours [19]. This may be due to differences in modelling strategies, the different periods of measurement used, or different data limitations which make individual studies uniquely susceptible to different forms of confounding. For example, of the three studies above only one controlled for the behaviours of parents or peers [17], while one was unable to account for individual level socioeconomic position [16]. This is relevant because there is strong evidence for the intergenerational transmission of smoking behaviour [20] and smoking behaviour is strongly associated with low socioeconomic position [21]. It is therefore likely that the associations between neighbourhood deprivation and health behaviours in these studies were biased by inadequate control of confounding variables. Because longitudinal studies can model temporal trends in exposure they are able to provide a full empirical account of neighbourhood history and explore how lifetime exposure to deprivation effects health. Despite this strength, there remains a lack of research that explicitly explores how long-term exposure to neighbourhood deprivation may impact upon health behaviours. It has been argued that neighbourhood of birth is important due to the problem of ‘stickiness’ [22]—that people tend to remain in the same types of neighbourhoods–but there has been little work examining how the temporal ordering of deprivation exposure matters for health outcomes beyond neighbourhood of birth. Analytical methods that employ a longitudinal approach and examine how different trajectories of exposures to deprivation impact upon health are required to further understanding of neighbourhood effects.

Inadequate controls may further exacerbate the non-random process of neighbourhood selection [23]. In general, wealthier and healthier individuals are more likely to move (select) into more affluent neighbourhoods than poorer and less healthy individuals [24]. This results in a non-random distribution of individuals across neighbourhoods that can introduce bias into analyses of neighbourhood effects. For example, an association between neighbourhood deprivation and smoking behaviour may represent an endogenous effect by which non-smokers select into affluent areas and smokers into deprived areas rather than the assumed exogenous effect of the neighbourhood acting upon people’s behaviour. The neighbourhood selection phenomenon makes it difficult to separate the ‘true’ contextual neighbourhood effects from compositional effects which reflect the make-up of a neighbourhood. Failing to account for neighbourhood selection may result in associations that appear to be contextual but are in fact compositional, and therefore impress a reverse causal association. This has been previously demonstrated by Jokela (2014), who found that the association between neighbourhood socioeconomic status and adult smoking reflected neighbourhood composition due to health selective migration rather than contextual neighbourhood effects [25]. Associations between neighbourhood deprivation and smoking were inflated because people who moved to poorer neighbourhoods were more likely to smoke [25].

We argue that a focus on children rather than adults offers several advantages for neighbourhood effects research into health behaviours such as smoking behaviour. First, health damaging behaviours are commonly encountered during adolescence, and once established can track into adulthood and persist throughout the later life course [26]. This makes the adolescent years an optimal time to intervene. Second, unlike adults, children are usually not the decision makers when it comes to family relocation [27]. While parents may select neighbourhoods because of attributes that they associate with successful childhood outcomes (such as school catchment areas in the UK), the process of selection is not one that the child actively participates in. This means that neighbourhood selection bias (and therefore reverse causality) is reduced because children do not select their destination neighbourhoods. Third, children have lower independent mobility than adults and are therefore more reliant on, and susceptible to, neighbourhood characteristics. That is, they are more likely to gain greater exposures to, and stronger effects from, their neighbourhood context. Fourth, adolescents are particularly susceptible to external influences in the development of behaviours because social factors at this stage of the life course are “the beginnings of socially determined pathways to health in adult life” [28].

We contribute to the neighbourhood effects literature in two distinct ways. First, we investigate whether associations between neighbourhood deprivation in childhood and cigarette smoking are due to compositional or contextual neighbourhood effects. We examine if families with parents who smoke are more likely to reside in deprived neighbourhoods, suggesting a compositional effect, or if neighbourhood effects operate above and beyond the family unit, suggesting a contextual effect. Second, we investigate the temporal ordering of neighbourhood deprivation throughout childhood to examine if a longitudinal approach to exposure improves upon analyses that use only neighbourhood of birth.

## Materials and methods

### 2.1 Study sample

**Fig 1:**
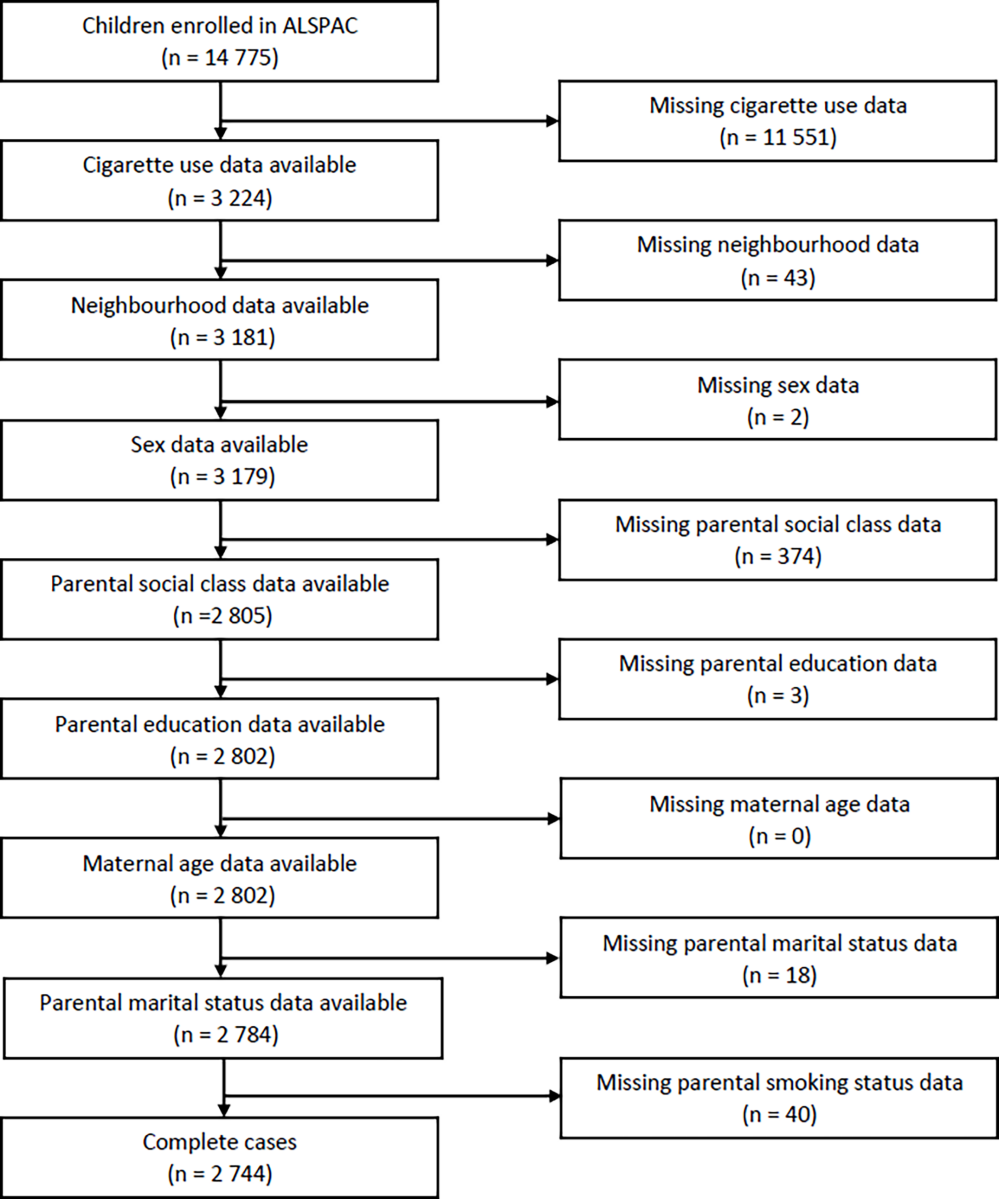
Attrition in the analytical sample.

We use data from the Avon Longitudinal Study of Parents and Children (ALSPAC). All pregnant women resident in the (former) Avon Health Authority area in South West England with an expected date of delivery between April 1991 and December 1992 were eligible to enrol. After birth, data were primarily collected from the study mothers and then from children via regular self-completion questionnaires and direct assessments. The ALSPAC cohort is largely representative of the UK population when compared with 1991 Census data; however, there is under representation of ethnic minorities, single parent families, and those living in rented accommodation. Ethical approval for the study was obtained from the ALSPAC Ethics and Law Committee and the Local Research Ethics Committees. For full details of the cohort profile and study design see [29,30]. Fig 1 shows the available analytical sample for our study and the causes of attrition. From the full enrolled sample of 14,775 children, 2,744 have full data and are used in our analyses.

### 2.2 Outcome variable

Our outcome variable was weekly number of cigarettes smoked by the study child at the age 17 “Teen Focus 4” direct assessment (median age: 17.67 years). Children self-reported their weekly cigarette consumption as part of a battery of questions on smoking habits with responses recorded on computer terminals in isolated rooms. The weekly cigarette outcome variable is therefore a count variable characterised by excess zeros where non-smokers are represented by the value of zero cigarettes per week. One important element that requires discussion relates to the legal restrictions around cigarette smoking in the UK. From 1^st^ October 2007 laws were brought into effect raising the legal age for cigarette purchase and consumption from 16 to 18. At the time of data collection 2,234 children (81.41%) were below the legal age of 18 and therefore reporting on illegal behaviour (although a number of these may have previously been legal smokers *prior* to the change in law). However, previous analysis on this highly engaged cohort has revealed that the reporting of illegal behaviour is higher in the privacy of direct assessment settings than in self-completed questionnaires [17], reducing the likelihood of measurement error attributable to misreporting.

### 2.3 Exposure variable

Our exposure variable is neighbourhood deprivation measured using quintiles of the 2004 UK Index of Multiple Deprivation (IMD) based on 2001 Lower Layer Super Output Areas (LSOA) (see [31] for detail)[31]. The IMD gives an objective score of socioeconomic deprivation for all neighbourhoods in the UK from least to most deprived. LSOAs are designed to be homogenous in size with an average population of 1500 residents, and are the finest scale at which the IMD is available. We use quintiles of the IMD and define neighbourhoods as being deprived if they are in the most deprived quintile of neighbourhoods in the UK. The 2004 IMD was used as it represents the midpoint of our study. Previous work has demonstrated that neighbourhood deprivation is consistent over time, meaning that our results should be robust to alternative measures of IMD [32]. Information on the neighbourhoods in which families lived was extracted from the ALSPAC address database, a data source used for cohort administrative purposes to record and track all addresses that study participants had lived in in order to maintain communication. Addresses were extracted at birth and then annually through to age 17, resulting in 18 measurements of neighbourhood deprivation based on the IMD.

To examine if longitudinal exposure to neighbourhood deprivation impacts upon smoking behaviour at age 17 we employ latent class analysis with trajectory models (LCA; see [33]). These models use repeat observation data to identify the major empirically distinct trajectory classes; in our case annual longitudinal exposures to neighbourhood deprivation from birth to age 17. Individuals are grouped together into these classes where their longitudinal trajectory of exposures is similar. We determine the number and shape of classes by comparing model fit criteria in keeping with best practice (see [34]). LCA offers a data driven approach to characterising the most common patterns of exposures to neighbourhood deprivation, enabling us to determine if certain neighbourhood trajectories are associated with smoking behaviour. LCA offers several advantages over pre-specified trajectories. First, because it is data driven it is robust to any researcher bias on potential patterns. Second, because trajectories of neighbourhood deprivation have not been commonly discussed in the literature they offer a sensible starting point to identifying the most common experiences amongst children. Given the data in the analytical dataset, four trajectory groups are obtained (Table 1). These trajectory groups are used as a categorical exposure variable to explain differences in smoking behaviour at age 17.

**Table 1:** Trajectory groups identified from the data.

Trajectory group name	Pattern of group longitudinal exposure to neighbourhood deprivation	Number in group	%
Stable non-deprived	*Spend the entirety of childhood in non-deprived neighbourhoods*, *experiencing no deprivation*.	2,416	88.05
Increasing deprivation	*Born into non-deprived neighbourhoods but transition to deprived neighbourhoods later in childhood*, *experiencing an increase in exposure to deprivation*	*35*	*1*.*28*
Declining deprivation	*Born into deprived neighbourhoods but transition to non-deprived neighbourhoods later in childhood*, *experiencing a decline in exposure to deprivation*	158	5.76
Stable Deprived	*Spend the entirety of childhood in deprived neighbourhoods*, *experiencing only deprivation*	135	4.92

### 2.4 Covariates

We use a range of covariates related to neighbourhood socioeconomic status and smoking behaviour. These include: child age in years at the age 17 direct assessment; sex of child and maternal age in years at birth; highest parental education (categorised as common certificate of education/none/vocational, O-level [exams taken at completion of compulsory school attendance], A-level [exams taken in post-compulsory schooling at age 18], and university degree or above) and highest parental Social Class based on Occupation (social classes I [Professional occupations], II [Managerial and technical occupations], and III-N [non-manual Skilled occupations] combined together into one category, and III-M [manual Skilled occupations], IV [Partly-skilled occupations], and V [Unskilled occupations] combined into another category) from the mothers self-report “Your Pregnancy” questionnaire during pregnancy; parental marriage status from the mothers self-report “Your Environment” questionnaire during pregnancy (categorised as married, partnered or single); and parental smoking habits (coded as no parents smoke, one parent smokes, or both parents smoke). Parental smoking habits were taken from responses to 10 self-report questionnaires from birth through to age 12 to boost sample size. These were: "Having A Baby", "Your Pregnancy" and "Being a Father" (father) during pregnancy; “Me And My Baby" at 8 weeks; "Looking After The Baby" and "The baby and me" (father) at one year; "Caring For A Toddler" and "A Toddler in the House" (father) at 2 years; "Mother's New Questionnaire" at 4 years; "Twelve years on" at age 12. The ALSPAC study website contains details of all the data that is available through a fully searchable data dictionary (http://www.bristol.ac.uk/alspac/researchers/access/).

### 2.5 Statistical analysis

Because our outcome is a count variable with excess zeroes, we use zero-inflated negative binomial (ZINB) models to measure the impact of neighbourhood deprivation on smoking behaviour. ZINB models partition the analysis into two separate elements on the underlying theory that there are two different processes generating responses that may be independent from one another. The first process is the likelihood that an individual is an excess zero, in our data a non-smoker, and is modelled using a logit function. The second process is the predicted count for a non-zero individual, or in our data, the number of cigarettes that a smoker is predicted to smoke on a weekly basis. This second process is modelled using a negative binomial function to account for over dispersion. This modelling approach allows us to simultaneously examine smoking status and smoking heaviness given exposure to neighbourhood deprivation. We use the same covariates in each part of the model as they are hypothesised to influence both processes. We present three sets of models throughout; unadjusted for covariates; adjusted for covariates; adjusted for covariates and parental smoking behaviour. This approach allows us to determine the extent to which observed associations between neighbourhood deprivation and cigarette smoking can be explained by (1) background socioeconomic and demographic characteristics, and (2) parental smoking behaviour. Robust standard errors clustered by neighbourhoods at age 17 were used to account for the clustering of individuals (mean: 3.2 participants per neighbourhood; range 1 to 14; 869 neighbourhoods represented) and resulting non-independence of residuals. Age 17 was chosen for clustering as this was the age at outcome variable measurement.

## Results

### 3.1 Descriptive statistics

Table 2 displays the descriptive statistics of our analytical sample. Of the 2,744 children retained after list wise deletion of missing data, 579 (21.1%) report smoking on a weekly basis. Amongst smokers the mean number of weekly cigarettes smoked was 45 (Interquartile Range: 15 to 70). 55.03% were female and the majority were born into families of higher socioeconomic position when measured by parental occupation and maternal education. Most of the sample were born into married families (85.24%), with only 4.41% born into single parent households. 62.35% of children grew up in families where neither parent smoked, 23.18% in families where one parent smoked, and 14.47% where both parents smoked. The median age at assessment was 17.75 years (IQR: 17.58 to 17.83). Differences exist between our analytical sample and the full ALSPAC cohort (Table A in S1 File), and we return to the potential implication of this in the discussion.

**Table 2:** Sample descriptive statistics.

	number	%
Smoker	579	21.10
Non-smoker	2,165	78.90
Female	1,510	55.03
Male	1,234	44.97
*Social class*		
I	539	19.64
II	1,270	46.28
III (non-manual)	588	21.43
III (manual)	248	9.04
IV	99	3.61
*Parental education*		
Degree	882	32.14
A-level	985	35.90
O-level	622	22.67
CSE/vocational	255	9.29
*Number of parents who smoke*		
Neither	1,711	62.35
One	636	23.18
Both	397	14.47
*Family status*		
Married	2,339	85.24
Partnered	284	10.35
Single	121	4.41
	mean	IQR
No. of cigarettes (smokers)	45.07	15 to 70
Maternal age at birth	29.57	27 to 32
Age at assessment	17.75	17.58 to 17.83

### 3.2 The influence of neighbourhood deprivation on smoking behaviour

Table 3 displays the association between neighbourhood deprivation at birth and cigarette smoking behaviour at age 17. The first part (‘smoker’) displays the Odds Ratios of a child born into neighbourhood deprivation quintiles 2–5 being a smoker at age 17 compared to a child who was born into the least deprived quintile of neighbourhoods. The second part (‘weekly cigarettes’) displays the log count of cigarettes that a smoker born into the same quintiles of neighbourhood deprivation is predicted to consume weekly compared to a child who was born into the least deprived quintile. The results from the unadjusted analysis, including only sex and age (Table 3, Model 1), show a positive association between neighbourhood deprivation at birth and likelihood of being a smoker at age 17. Overall, the higher the level of neighbourhood deprivation that a child is born into the more likely they are to smoke. Those born into families living in the most deprived neighbourhood quintile are twice as likely (Odds Ratio: 1.94; 95% Confidence Interval: 1.37 to 2.74) to be a smoker as those born into the least deprived quintile of neighbourhoods. Turning to the second part of the model, there is a positive association between neighbourhood deprivation and the number of cigarettes consumed. Taking the exponentiation of the coefficient 0.216, children born into the most deprived neighbourhoods are expected to smoke 24.1% (95% CI: -2.1 to 57.3) more cigarettes than those born into the least deprived quintile of neighbourhoods, however the noise around this point estimate limits the strength of this evidence. The lack of coefficient precision in the count part of the model may be due to the reduced sample size of smokers (n = 579).

**Table 3:** Neighbourhood deprivation at birth and smoking behaviour at age 17.

	Model 1: Unadjusted			Model 2: SEP adjusted			Model 3: Parental smoking adjusted		
	OR/Coef.	95% CI	p value	OR/Coef.	95% CI	p value	OR/Coef.	95% CI	p value
*Smoker*									
Q1 –least deprived	Ref			Ref			Ref		
Q2	1.418	1.075 to 1.870	0.014	1.377	1.041 to 1.822	0.025	1.305	0.983 to 1.733	0.066
Q3	1.365	1.040 to 1.793	0.025	1.251	0.947 to 1.650	0.114	1.200	0.904 to 1.592	0.207
Q4	1.419	1.028 to 1.960	0.033	1.262	0.908 to 1.756	0.167	1.151	0.824 to 1.610	0.409
Q5	1.937	1.372 to 2.735	<0.001	1.559	1.088 to 2.234	0.016	1.223	0.848 to 1.765	0.282
Constant	0.218	0.175 to 0.270	<0.001	0.189	0.083 to 0.432	<0.001	0.117	0.051 to 0.270	<0.001
*Weekly cigarettes*								
Q1 –least deprived	Ref			Ref			Ref		
Q2	0.018	-0.211 to 0.247	0.877	0.032	-0.206 to 0.269	0.794	-0.018	-0.267 to 0.232	0.890
Q3	0.031	-0.186 to 0.248	0.781	-0.062	-0.269 to 0.144	0.554	-0.093	-0.302 to 0.116	0.382
Q4	0.109	-0.124 to 0.341	0.359	0.039	-0.206 to 0.284	0.756	-0.003	-0.257 to 0.251	0.982
Q5	0.216	-0.021 to 0.453	0.074	0.021	-0.236 to 0.278	0.874	-0.052	-0.318 to 0.214	0.701
Constant	3.685	3.505 to 3.864	<0.001	3.890	3.238 to 4.542	<0.001	3.711	3.056 to 4.366	<0.001

OR, Odds Ratio; Coef, Coefficient; CI, confidence interval; Q1, quintile 1; Ref, reference category. Model 2 is adjusted for age, sex, parental social class, highest parental education, maternal age and marital status. Model 3 is additionally adjusted for number of parents who smoke. For full model results see Tables B-D in S1 File.

To test the extent to which the association between neighbourhood deprivation at birth and smoking behaviour at age 17 is a function of family socio-economic position, Model 2 in Table 3 includes controls for parental occupational social class, maternal education, maternal age at birth, and parental relationship at birth. These covariates have a strong attenuating effect on the association between neighbourhood deprivation and smoking behaviour, suggesting that the relationship between deprivation and smoking is in part a function of family socioeconomic position. Associations remain between neighbourhood deprivation and likelihood of being a smoker for children born into the second and most deprived quintiles of deprivation. Those born into the most deprived quintile are 55.9% (95% CI: 8.8 to 123.4) more likely to be smokers at age 17 than children born into the least deprived quintile. All statistical support for the association between neighbourhood deprivation and smoking heaviness for children born into the most deprived quintile of neighbourhoods is removed, with smokers in these areas predicted to smoke only 2.1% (95% CI: -21.2 to 32.0) more cigarettes than children from the least deprived areas.

One potential social sorting mechanism that may be confounding these results is that of parental smoking behaviour. To address this issue Model 3 in Table 3 additionally controls for parental smoking behaviour. Odds Ratios are further attenuated though evidence for an association remains whereby children born into the second least deprived quintile of neighbourhoods are more likely to be smokers than those in the least deprived quintile. These findings suggest that confounding by parental smoking behaviour upwardly biases the association between neighbourhood deprivation and smoking behaviour.

### 3.3 Changes to neighbourhood deprivation

The analysis thus far has examined only the impact of neighbourhood deprivation *at birth* on smoking behaviour at age 17 and therefore ignores the influence of *lifetime* exposure to neighbourhood deprivation. Where children change neighbourhood, their exposure to deprivation will be incorrectly apportioned to the neighbourhood of birth only. To examine the impact of longitudinal experience of neighbourhood deprivation upon smoking behaviour we replace neighbourhood deprivation at birth in the models with trajectories of deprivation (Table 4). Compared to children who remain in non-deprived neighbourhoods throughout the entirety of their childhood, children who transition from deprived to non-deprived neighbourhoods and those who remain in deprived neighbourhoods are 54.2% (95% CI: 6.1 to 124.3) and 53.9% (95% CI: 3.10 to 129.6) more likely to be smokers respectively (Table 4 Model 1). The results are similar for the number of cigarettes that a given smoker is predicted to smoke, with those who transition from deprived to non-deprived neighbourhoods predicted to smoke 36.5% (95% CI: 6.7 to 74.4) more cigarettes and those who remain in deprived neighbourhoods predicted to smoke 33.0% (95% CI: 10.2 to 60.3) more cigarettes than children who remain in non-deprived neighbourhoods. There is no evidence for a difference in smoking behaviour between children who experience a rise in neighbourhood deprivation and those who remain in non-deprived neighbourhoods, however this is likely due to low numbers in this group (n = 35).

**Table 4:** Longitudinal neighbourhood deprivation transitions and smoking behaviour at age 17.

	Model 1: Unadjusted			Model 2: SEP adjusted			Model 3: Parental smoking adjusted		
	OR/Coef.	95% CI	p value	OR/Coef.	95% CI	p value	OR/Coef.	95% CI	p value
*Smoker*									
*Stable non-deprived*	*Ref*			*Ref*			*Ref*		*Ref*
*Rising deprivation*	1.317	0.641 to 2.702	0.453	1.093	0.531 to 2.248	0.81	0.945	0.463 to 1.923	0.875
*Declining deprivation*	1.542	1.061 to 2.243	0.023	1.274	0.859 to 1.887	0.229	1.073	0.727 to 1.584	0.723
*Stable deprived*	1.539	1.031 to 2.296	0.035	1.245	0.827 to 1.876	0.294	1.027	0.667 to 1.582	0.902
Constant	0.279	0.243 to 0.320	<0.001	0.223	0.101 to 0.491	<0.001	0.143	0.065 to 0.317	<0.001
*Weekly cigarettes*									
*Stable non-deprived*	*Ref*			*Ref*			*Ref*		*Ref*
*Rising deprivation*	0.100	-0.148 to 0.348	0.431	-0.121	-0.373 to 0.131	0.348	-0.171	-0.412 to 0.07	0.164
*Declining deprivation*	0.311	0.065 to 0.556	0.013	0.155	-0.132 to 0.442	0.290	0.115	-0.184 to 0.414	0.450
*Stable deprived*	0.285	0.097 to 0.472	0.003	0.094	-0.101 to 0.290	0.343	0.082	-0.114 to 0.279	0.412
Constant	3.704	3.600 to 3.808	<0.001	3.861	3.269 to 4.453	<0.001	3.712	3.115 to 4.308	<0.001

OR, Odds Ratio; Coef, Coefficient; CI, confidence interval; Q1, quintile 1; Ref, reference category. Model 2 is adjusted for age, sex, parental social class, highest parental education, maternal age and marital status. Model 3 is additionally adjusted for number of parents who smoke. For full model results see Tables E-G in S1 File.

The results adjusted for family socioeconomic and demographic covariates (Table 4, Model 2) demonstrate that the associations between trajectory groups and smoking behaviour are largely a function of family socioeconomic position rather than direct neighbourhood effects. Statistical support for all associations is removed and Odds Ratios are attenuated. This suggests that the unadjusted associations were confounded by family socioeconomic position. Adjusting for parental smoking behaviour (Table 4 Model 3) further attenuates associations, demonstrating that the associations between trajectory groups and smoking behaviour are also a function of parental smoking behaviour.

### 3.4 Relative deprivation and residential mobility

**Fig 2:**
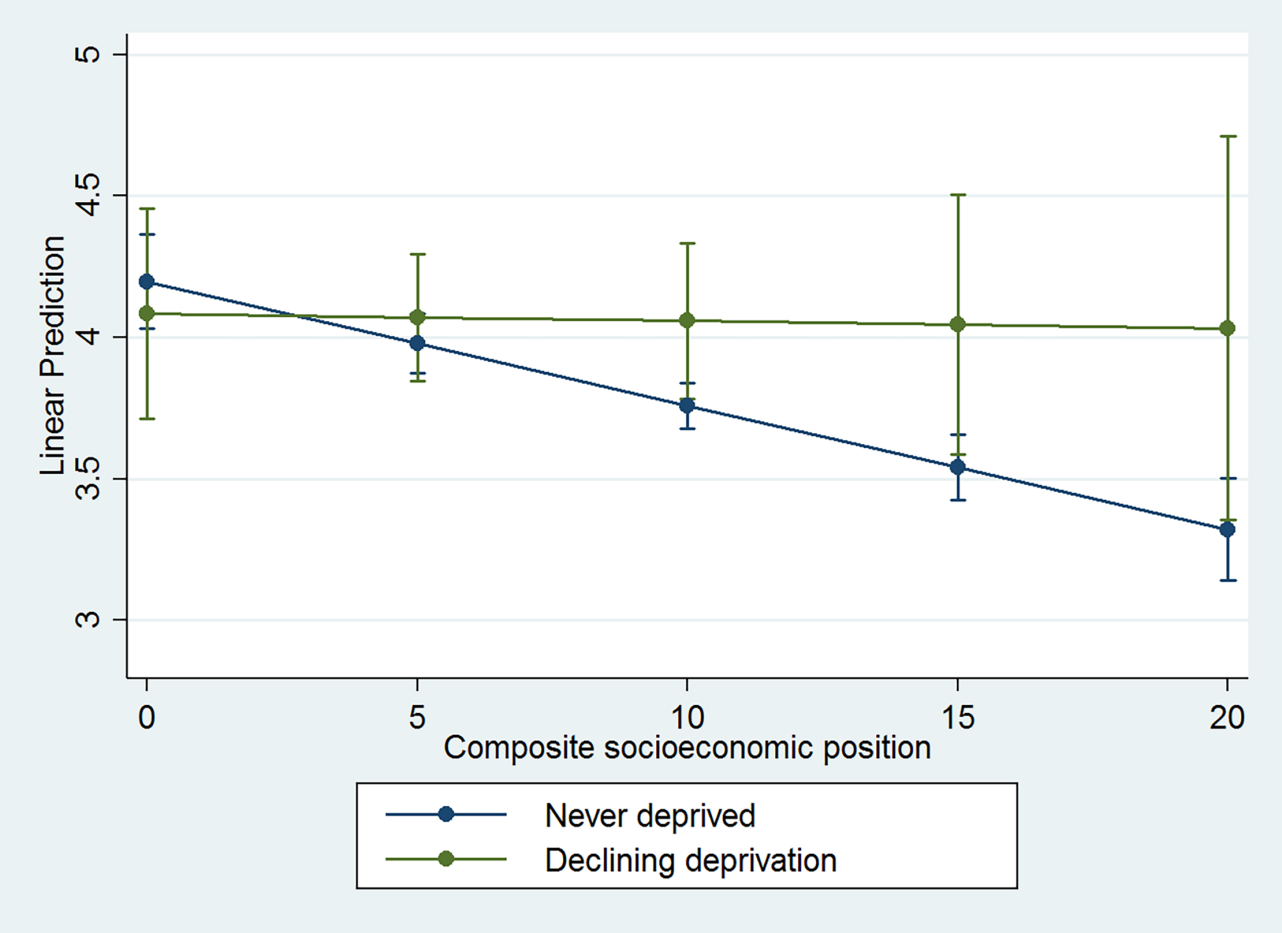
Linear prediction of interaction effect between neighbourhood deprivation and composite family socioeconomic position on number of cigarettes smoked at age 17. For full model results see Table H in S1 File.

The results reveal a counterintuitive finding whereby children who experience a decline in neighbourhood deprivation have the poorest smoking outcomes. We investigated if this was a consequence of relative deprivation, the phenomenon whereby an individual who is affluent in absolute terms may be deprived relative to their surroundings or peers. This has been hypothesised as a potential mechanism for explaining negative outcomes amongst individuals who make positive moves away from deprived neighbourhoods [35]. We combined data on parental education and social class to create a composite indicator of family socioeconomic position and interacted this with the neighbourhood trajectories. The results (Fig 2) show that while the slopes differ between the declining neighbourhood deprivation and the never-deprived groups, there is no statistical evidence for an interaction effect. That is, relative deprivation is not responsible for this finding.

Another possible reason is the potentially disruptive experience of moving to a new house that will accompany a change of neighbourhood. 51% of families in ALSPAC move to a new house in the period that our analysis covers and many of these will experience a change in neighbourhood [36]. We examined whether the number of household moves a child’s family had made during the study period impacted their smoking behaviour over and above the trajectory groups. The results of this analysis (Table 5) show an independent association between the number of household moves and a child’s likelihood of smoking cigarettes at age 17. For each additional move the likelihood of being a smoker increases by 12.1% (95% CI: 3.40% to 21.4%), suggesting that increased levels of residential mobility throughout childhood may have a negative impact upon smoking behaviour. There was no evidence for an association between moving and smoking heaviness. The inclusion of household moves in the model also has an impact on the results of the trajectory groups. First, in the logit part of the model the point estimate for the stable deprived group is considerably higher at 1.57 (95% CI: 1.05 to 2.35) than for the declining deprivation group at 1.36 (95% CI: 0.93 to 1.99). This, combined with the independent effect of household moves, suggests that the (counterintuitive) elevated risk of being in the declining deprivation group in Table 4 is in part capturing the negative impact of moving house (which accompanies a neighbourhood transition) on likelihood of cigarette smoking. Second, in the count part of the model the point estimate for the declining deprivation group is now comparable to that of the stable deprived group, further reinforcing the possibility that the results in Table 4 may have been biased by residential mobility.

**Table 5:** Longitudinal neighbourhood deprivation transitions and smoking behaviour at age 17, adjusted for household moves.

	Model 1: Unadjusted			Model 2: SEP adjusted			Model 3: Parental smoking adjusted		
	OR/Coef.	95% CI	p value	OR/Coef.	95% CI	p value	OR/Coef.	95% CI	p value
*Smoker*									
*Stable non-deprived*	*Ref*	*Ref*	*Ref*	*Ref*	*Ref*	*Ref*	*Ref*	*Ref*	*Ref*
*Rising deprivation*	1.105	0.530 to 2.303	0.789	0.955	0.458 to 1.990	0.902	0.885	0.427 to 1.837	0.743
*Declining deprivation*	1.359	0.929 to 1.988	0.114	1.169	0.787 to 1.735	0.438	1.031	0.697 to 1.528	0.876
*Stable deprived*	1.571	1.053 to 2.347	0.027	1.283	0.849 to 1.937	0.237	1.041	0.674 to 1.610	0.856
Household move	1.121	1.034 to 1.214	0.006	1.104	1.013 to 1.203	0.024	1.047	0.961 to 1.141	0.299
Constant	0.247	0.209 to 0.293	<0.001	0.160	0.068 to 0.380	<0.001	0.121	0.051 to 0.289	<0.001
*Weekly cigarettes*									
*Stable non-deprived*	*Ref*	*Ref*	*Ref*	*Ref*	*Ref*	*Ref*	*Ref*	*Ref*	*Ref*
*Rising deprivation*	0.075	-0.198 to 0.348	0.590	-0.111	-0.390 to 0.167	0.433	-0.139	-0.410 to 0.132	0.315
*Declining deprivation*	0.290	0.029 to 0.551	0.029	0.161	-0.142 to 0.464	0.299	0.134	-0.174 to 0.443	0.393
*Stable deprived*	0.284	0.096 to 0.472	0.003	0.094	-0.101 to 0.289	0.344	0.080	-0.116 to 0.277	0.423
Household move	0.013	-0.036 to 0.063	0.595	-0.005	-0.056 to 0.046	0.853	-0.018	-0.071 to 0.035	0.509
Constant	3.688	3.567 to 3.808	<0.001	3.877	3.265 to 4.488	<0.001	3.764	3.154 to 4.373	<0.001

OR, Odds Ratio; Coef, Coefficient; CI, confidence interval; Q1, quintile 1; Ref, reference category. Model 2 is adjusted for age, sex, parental social class, highest parental education, maternal age and marital status. Model 3 is additionally adjusted for number of parents who smoke. For full model results see Tables I-K in S1 File.

## Discussion

In this study, we find a positive association between neighbourhood deprivation and smoking behaviour at age 17. Children born into more socioeconomically deprived neighbourhoods are up to twice as likely to go on to become smokers than those born into less deprived neighbourhoods. There is suggestive evidence that those born into the most deprived quintile of neighbourhoods are also likely to be heavier smokers, with a 25% higher cigarettes consumption than those born into the least deprived quintile. These associations are largely a function of family socioeconomic position and parental smoking behaviour, whereby children born into deprived neighbourhoods are more likely to have parents who smoke which in turn will increase their likelihood of smoking. As such, the development of adolescent smoking behaviour in this sample is due to compositional rather than direct contextual neighbourhood effects; it is not the deprived neighbourhoods themselves that give rise to deleterious smoking behaviours, but the composition of people residing within them. Individual level circumstance reflects intergenerational transmission of smoking behaviour from parents to children and confounds the association between neighbourhood deprivation and smoking. This substantiates previous research showing that individual socioeconomic factors explain associations between neighbourhood deprivation and health behaviours in part or in full [18,37,38], and supports a family level social selection mechanism as has been previously demonstrated in this context [25]. It is possible that these results reflect upstream neighbourhood effects that act upon parents, which in turn operate on children; a phenomenon that may represent an ‘indirect contextual effect’. Future studies exploring how compositional or contextual neighbourhood effects on children may operate via parents may help to elucidate this.

Our findings also highlight the importance of considering longitudinal trajectories of exposure to neighbourhood deprivation when examining health damaging behaviours such as cigarette smoking. Compared to individuals who experience no exposure to neighbourhood deprivation throughout childhood, individuals who are born into deprived neighbourhoods are more likely to be smokers and smoke heavily at age 17, regardless of whether they subsequently stay in deprived neighbourhoods or move into non-deprived neighbourhoods. These associations are largely a function of family socioeconomic status and parental smoking behaviour, providing longitudinal support for a social sorting mechanism rather than a direct impact of neighbourhood deprivation on smoking behaviour. These results corroborate those from another study which found no direct effect of neighbourhood socioeconomic status on people who changed neighbourhoods [16]. Furthermore, our results are consistent with those exploring neighbourhood deprivation and associated factors with adolescent smoking from US experimental settings such as the Yonkers Family and Community Survey and the Moving To Opportunity experiment [39–41]. While these studies are drawn from a different national sample to ALSPAC and therefore their results may not be directly comparable, their quasi-experimental designs provide a stronger framework for casual inference. Weak statistical power leads to a lack of imprecision in point estimates for children who experience an increase in deprivation throughout childhood, and future studies with larger samples of children who transition into more deprived neighbourhoods may be able to more precisely identify associations. Interestingly we find that children who make transitions from deprived to non-deprived neighbourhoods have poorer outcomes than those who remain in deprived neighbourhoods throughout childhood. We find no evidence that this is due to relative deprivation, though previous studies have suggested this [18]. Instead, this counterintuitive finding can be partly explained by the negative impact that residential mobility has on cigarette smoking. This supports previous findings on the ALSPAC cohort that residential moves were more important than changes in neighbourhood deprivation for adolescent cannabis use [17]. It is also possible that children who make moves to less deprived neighbourhoods fall back into old behaviours or peer groups as has been previously suggested [42], although we were unable to test this given the data.

Several limitations exist with this study. First, because we used a binary measure of neighbourhood deprivation at the fifth quintile to determine neighbourhood deprivation we were unable to test if changes in the level of deprivation had an influence on a child’s smoking behaviour. This is a common limitation amongst studies such as ours and cannot be avoided when using cut-offs to define a neighbourhood as deprived. Second, our measure of deprivation was extracted at only a single point in time based upon the 2004 IMD. This means that neighbourhood values of deprivation were constrained over time and therefore assumes no change in deprivation. The socioeconomic characteristics of neighbourhoods upon which the IMD are built change with time, and as a result the classification and ordering of neighbourhoods will also change. However, previous work has revealed that neighbourhood deprivation is generally stable over time [32] and this is unlikely to cause considerable bias in our results. Third, our sample of smokers was small compared to our total sample, which reduced the statistical power with which we could identify associations on smoking heaviness. Future studies with larger samples of adolescent smokers may be able to elaborate upon our findings and more accurately identify potential neighbourhood effects. Fourth, due to data limitations we were unable to disentangle residential neighbourhood from school effects and therefore our results could capture school level factors. This is an area that future studies may be able to explore. Finally, as with almost all longitudinal studies it is possible that our results may be biased by cohort attrition. As a result, our findings may not be generalisable to the full cohort (or indeed the wider UK population). However, as we are estimating associations between neighbourhood deprivation and adolescent cigarette smoking instead of population distributions, complex forms of associational drop out would be required to invalidate our findings [43].

In conclusion, we find that children who are born into or grow up in deprived neighbourhoods are more likely to be smokers at age 17 than those in non-deprived neighbourhoods. These associations however are largely due to family socioeconomic position and the intergenerational transmission of smoking behaviour from parents to children, rather than direct contextual ‘neighbourhood effects’. It is not so much where a child is born or lives, but who they are born to that determines their smoking behaviour upon entering adulthood.

## Supporting information

S1 File Supporting information for main manuscript. (DOCX)
