# Supplementary material for: Context or composition: How does neighbourhood deprivation impact upon adolescent smoking behaviour?
Source: PLoS One. 2018 Feb 8;13(2):e0192566. doi: 10.1371/journal.pone.0192566 (PMC5805312; doi:10.1371/journal.pone.0192566)
Supplement: S1 File — (DOCX) [file pone.0192566.s001.docx]

# S1 File. Supplementary material to “Context or composition: How does neighbourhood deprivation impact upon adolescent smoking behaviour?”

Table S1 displays differences between the analytical sample and the full ALSPAC cohort. Numbers differ across variables due to differing number of responses to each in the cohort: these analyses each utilise the maximum sample available regardless of other variables. Children in the analytical sample come from families that: are of higher social class; have more highly educated parents; are more likely to be headed by married parents; have parents who are less likely to smoke; and whose mothers are older. It is possible that the differences between our analytical sample and the wider cohort may represent selection bias into our study and lead to bias in the estimates. However, because we are interested in associations between neighbourhood deprivation and adolescent cigarette smoking, not data on average levels or the distribution of factors across the population, attrition should not invalidate our findings. Complex forms of associational drop out are required for attrition or non-representativeness to invalidate associations (Rothman et al, 2013).

Rothman KJ, Gallacher JE, Hatch EE. 2013. Why representativeness should be avoided. *Int J Epidemiol* 42(4):1012-4. doi: 10.1093/ije/dys223.

## Table A: Differences between analytical sample and full cohort

|  | Excluded | |  | Included |  | p value for difference |
| --- | --- | --- | --- | --- | --- | --- |
|  | n | | Column % | n | Column % |  |
| *Sex* |  | |  |  |  | <0.001 |
| Male | 6,398 | | 52.85 | 1,234 | 44.97 |  |
| Female | 5,709 | | 47.15 | 1,510 | 55.03 |  |
|  |  | |  |  |  |  |
| *Parental social class* | |  | |  |  | <0.001 |
| I | 1001 | | 11.34 | 539 | 19.64 |  |
| II | 3,565 | | 40.38 | 1,270 | 46.28 |  |
| III-NM | 2,360 | | 26.73 | 588 | 21.43 |  |
| III-M | 1,316 | | 14.91 | 248 | 9.04 |  |
| IV/V | 586 | | 6.64 | 99 | 3.61 |  |
|  |  | |  |  |  |  |
| *Parental education* | |  | |  |  | <0.001 |
| CSE | 1,573 | | 16.09 | 148 | 5.39 |  |
| Vocational | 723 | | 7.39 | 107 | 3.90 |  |
| O-level | 2,716 | | 27.78 | 622 | 22.67 |  |
| A-level | 2,985 | | 30.53 | 985 | 35.90 |  |
| Degree | 1,780 | | 18.21 | 882 | 32.14 |  |
|  |  | |  |  |  |  |
| *Parental marital status* | | | |  |  | <0.001 |
| Married | 7,806 | | 71.98 | 2,339 | 85.24 |  |
| Partnered | 2,069 | | 19.08 | 284 | 10.35 |  |
| Single | 969 | | 8.94 | 121 | 4.41 |  |
|  |  | |  |  |  |  |
| *Parental smoking* | |  | |  |  | <0.001 |
| Neither | 4,755 | | 46.18 | 1,711 | 62.35 |  |
| 1 | 2,972 | | 28.87 | 636 | 23.18 |  |
| 2 | 2,569 | | 24.95 | 397 | 14.47 |  |
|  |  | |  |  |  |  |
|  | mean | | SD | mean | SD |  |
| *Maternal age* | 27.62 | | 5.02 | 29.57 | 4.41 | <0.001 |

## Table B: Full model results from Table 2 Model 1 in main manuscript

|  | OR/Coef. | 95% CI | | p value | |  |
| --- | --- | --- | --- | --- | --- | --- |
| *Smoker* |  |  | |  | |  |
| Neighbourhood deprivation |  |  | |  | |  |
| *Q1 – least deprived* |  |  | |  | |  |
| *Q2* | 1.418 | 1.075 to 1.870 | | 0.014 | |  |
| *Q3* | 1.365 | 1.040 to 1.793 | | 0.025 | |  |
| *Q4* | 1.419 | 1.028 to 1.960 | | 0.033 | |  |
| *Q5 – most deprived* | 1.937 | 1.372 to 2.735 | | <0.001 | |  |
| Age | 1.413 | 1.092 to 1.828 | | 0.009 | |  |
| Male | 0.854 | 0.702 to 1.040 | | 0.116 | |  |
| Constant | 0.218 | 0.175 to 0.270 | | <0.001 | |  |
|  |  |  | |  | |  |
| *Weekly cigarettes* | | |  | |  | |
| Neighbourhood deprivation |  |  | |  | |  |
| *Q1 – least deprived* |  |  | |  | |  |
| *Q2* | 0.018 | -0.211 to 0.247 | | 0.877 | |  |
| *Q3* | 0.031 | -0.186 to 0.248 | | 0.781 | |  |
| *Q4* | 0.109 | -0.124 to 0.341 | | 0.359 | |  |
| *Q5 – most deprived* | 0.216 | -0.021 to 0.453 | | 0.074 | |  |
| Age | -0.048 | -0.250 to 0.154 | | 0.641 | |  |
| Male | 0.081 | -0.072 to 0.234 | | 0.299 | |  |
| Constant | 3.685 | 3.505 to 3.864 | | <0.001 | |  |
|  |  |  | |  | |  |
| /lnalpha | -0.095 | -0.231 to 0.040 | | 0.167 | |  |
| alpha | 0.909 | 0.794 to 1.041 | | <0.001 | |  |

OR, Odds Ratio; Coef, Coefficient; CI, confidence interval; Q1, quintile 1; Ref, reference category.

## Table C: Full model results from Table 2 Model 2 in main manuscript

|  | OR/Coef. | 95% CI | p value |
| --- | --- | --- | --- |
| Smoker |  |  |  |
| Neighbourhood deprivation |  |  |  |
| *Q1 – least deprived* |  |  |  |
| *Q2* | 1.377 | 1.041 to 1.822 | 0.025 |
| *Q3* | 1.251 | 0.947 to 1.650 | 0.114 |
| *Q4* | 1.262 | 0.908 to 1.756 | 0.167 |
| *Q5 – most deprived* | 1.559 | 1.088 to 2.234 | 0.016 |
| Age | 1.374 | 1.060 to 1.781 | 0.016 |
| Male | 0.872 | 0.715 to 1.063 | 0.175 |
| Parental social class |  |  |  |
| *I* | Ref. | Ref. | Ref. |
| *II* | 1.534 | 1.130 to 2.083 | 0.006 |
| *III (non-manual)* | 1.448 | 0.991 to 2.115 | 0.056 |
| *III (manual)* | 1.815 | 1.181 to 2.790 | 0.007 |
| *IV* | 1.874 | 1.069 to 3.284 | 0.028 |
| Parental education |  |  |  |
| *Degree* | Ref. | Ref. | Ref. |
| *A level* | 0.914 | 0.703 to 1.189 | 0.503 |
| *O level* | 1.027 | 0.754 to 1.398 | 0.865 |
| *CSE/vocational* | 1.274 | 0.856 to 1.896 | 0.233 |
| Maternal age | 0.993 | 0.969 to 1.018 | 0.578 |
| Parental Marriage status |  |  |  |
| *Married* | Ref. | Ref. | Ref. |
| *Partnered* | 1.145 | 0.834 to 1.571 | 0.401 |
| *Single* | 1.542 | 1.025 to 2.319 | 0.037 |
| Constant | 0.189 | 0.083 to 0.432 | <0.001 |
|  |  |  |  |
| *Weekly cigarettes* | |  |  |
| Neighbourhood deprivation |  |  |  |
| Q1 – least deprived |  |  |  |
| Q2 | 0.032 | -0.206 to 0.269 | 0.794 |
| Q3 | -0.062 | -0.269 to 0.144 | 0.554 |
| Q4 | 0.039 | -0.206 to 0.284 | 0.756 |
| Q5 – most deprived | 0.021 | -0.236 to 0.278 | 0.874 |
| Age | -0.072 | -0.276 to 0.132 | 0.491 |
| Male | 0.151 | -0.005 to 0.306 | 0.058 |
| Parental social class |  |  |  |
| *I* | Ref. | Ref. | Ref. |
| *II* | 0.220 | -0.081 to 0.521 | 0.153 |
| *III (non-manual)* | 0.299 | -0.044 to 0.642 | 0.088 |
| *III (manual)* | 0.354 | -0.001 to 0.709 | 0.051 |
| *IV* | 0.346 | -0.075 to 0.768 | 0.107 |
| Parental education |  |  |  |
| *Degree* | Ref. | Ref. | Ref. |
| *A level* | 0.075 | -0.164 to 0.314 | 0.538 |
| *O level* | 0.296 | 0.045 to 0.548 | 0.021 |
| *CSE/vocational* | 0.245 | -0.056 to 0.545 | 0.110 |
| Maternal age | -0.021 | -0.039 to -0.004 | 0.019 |
| Parental Marriage status |  |  |  |
| *Married* | Ref. | Ref. | Ref. |
| *Partnered* | 0.229 | 0.012 to 0.446 | 0.038 |
| *Single* | 0.313 | -0.018 to 0.643 | 0.064 |
| Constant | 3.890 | 3.238 to 4.542 | <0.001 |
|  |  |  |  |
| /lnalpha | -0.179 | -0.314 to -0.043 | 0.010 |
| alpha | 0.836 | 0.730 to 0.958 | <0.001 |

OR, Odds Ratio; Coef, Coefficient; CI, confidence interval; Q1, quintile 1; Ref, reference category.

## Table D: Full model results from Table 2 Model 3 in main manuscript

|  | OR/Coef. | 95% CI | p value |
| --- | --- | --- | --- |
| *Smoker* |  |  |  |
| Neighbourhood deprivation |  |  |  |
| *Q1 – least deprived* | Ref. | Ref. | Ref. |
| *Q2* | 1.305 | 0.983 to 1.733 | 0.066 |
| *Q3* | 1.200 | 0.904 to 1.592 | 0.207 |
| *Q4* | 1.151 | 0.824 to 1.610 | 0.409 |
| *Q5 – most deprived* | 1.223 | 0.848 to 1.765 | 0.282 |
| Age | 1.384 | 1.060 to 1.808 | 0.017 |
| Male | 0.851 | 0.696 to 1.040 | 0.114 |
| Parental social class |  |  |  |
| *I* | Ref. | Ref. | Ref. |
| *II* | 1.428 | 1.044 to 1.952 | 0.026 |
| *III (non-manual)* | 1.383 | 0.941 to 2.032 | 0.099 |
| *III (manual)* | 1.576 | 1.017 to 2.442 | 0.042 |
| *IV* | 1.690 | 0.953 to 2.995 | 0.072 |
| Parental education |  |  |  |
| *Degree* | Ref. | Ref. | Ref. |
| *A level* | 0.853 | 0.652 to 1.116 | 0.248 |
| *O level* | 0.931 | 0.680 to 1.275 | 0.657 |
| *CSE/vocational* | 1.043 | 0.691 to 1.573 | 0.842 |
| Maternal age | 1.005 | 0.979 to 1.030 | 0.721 |
| Parental Marriage status |  |  |  |
| *Married* | Ref. | Ref. | Ref. |
| *Partnered* | 0.882 | 0.631 to 1.234 | 0.465 |
| *Single* | 1.189 | 0.774 to 1.828 | 0.428 |
| Number of parents who smoke |  |  |  |
| Neither parent | Ref. | Ref. | Ref. |
| One parent | 1.837 | 1.436 to 2.347 | <0.001 |
| Both parents | 3.543 | 2.726 to 4.604 | <0.001 |
| Constant | 0.117 | 0.051 to 0.270 | <0.001 |
|  |  |  |  |
| *Weekly cigarettes* |  |  |  |
| Neighbourhood deprivation |  |  |  |
| *Q1 – least deprived* | Ref. | Ref. | Ref. |
| *Q2* | -0.018 | -0.267 to 0.232 | 0.890 |
| *Q3* | -0.093 | -0.302 to 0.116 | 0.382 |
| *Q4* | -0.003 | -0.257 to 0.251 | 0.982 |
| *Q5 – most deprived* | -0.052 | -0.318 to 0.214 | 0.701 |
| Age | -0.040 | -0.255 to 0.174 | 0.713 |
| Male | 0.160 | 0.004 to 0.317 | 0.045 |
| Parental social class |  |  |  |
| *I* | Ref. | Ref. | Ref. |
| *II* | 0.197 | -0.095 to 0.490 | 0.186 |
| *III (non-manual)* | 0.258 | -0.084 to 0.600 | 0.139 |
| *III (manual)* | 0.297 | -0.056 to 0.650 | 0.099 |
| *IV* | 0.301 | -0.112 to 0.714 | 0.153 |
| Parental education |  |  |  |
| *Degree* | Ref. | Ref. | Ref. |
| *A level* | 0.078 | -0.152 to 0.307 | 0.508 |
| *O level* | 0.290 | 0.042 to 0.539 | 0.022 |
| *CSE/vocational* | 0.227 | -0.072 to 0.526 | 0.137 |
| Maternal age | -0.017 | -0.035 to 0.000 | 0.053 |
| Parental Marriage status |  |  |  |
| *Married* | Ref. | Ref. | Ref. |
| *Partnered* | 0.211 | -0.013 to 0.434 | 0.064 |
| *Single* | 0.314 | -0.028 to 0.656 | 0.072 |
| Number of parents who smoke |  |  |  |
| Neither parent | Ref. | Ref. | Ref. |
| One parent | 0.202 | 0.008 to 0.395 | 0.041 |
| Both parents | 0.249 | 0.047 to 0.451 | 0.016 |
| Constant | 3.711 | 3.056 to 4.366 | <0.001 |
|  |  |  |  |
| /lnalpha | 1.212 | 1.058 to 1.388 | 0.006 |
| alpha | 0.438 | 0.389 to 0.487 | <0.001 |

OR, Odds Ratio; Coef, Coefficient; CI, confidence interval; Q1, quintile 1; Ref, reference category.

## Table E: Full model results from Table 3 Model 1 in main manuscript

|  | OR/Coef. | 95% CI | p value |
| --- | --- | --- | --- |
| *Smoker* |  |  |  |
| *Stable non-deprived* | *Ref* | *Ref* | *Ref* |
| *Rising deprivation* | 1.317 | 0.641 to 2.702 | 0.453 |
| *Declining deprivation* | 1.542 | 1.061 to 2.243 | 0.023 |
| *Stable deprived* | 1.539 | 1.031 to 2.296 | 0.035 |
| Age | 1.358 | 1.061 to 1.738 | 0.015 |
| Male | 0.832 | 0.689 to 1.005 | 0.057 |
| Constant | 0.279 | 0.243 to 0.320 | <0.001 |
|  |  |  |  |
| *Weekly cigarettes* | |  |  |
| *Stable non-deprived* | *Ref* | *Ref* | *Ref* |
| *Rising deprivation* | 0.100 | -0.148 to 0.348 | 0.431 |
| *Declining deprivation* | 0.311 | 0.065 to 0.556 | 0.013 |
| *Stable deprived* | 0.285 | 0.097 to 0.472 | 0.003 |
| Age | -0.057 | -0.251 to 0.136 | 0.56 |
| Male | 0.094 | -0.051 to 0.238 | 0.204 |
| Constant | 3.704 | 3.600 to 3.808 | <0.001 |
|  |  |  |  |
| /lnalpha | -0.109 | -0.239 to 0.022 | 0.102 |
| alpha | 0.897 | 0.787 to 1.022 | <0.001 |

OR, Odds Ratio; Coef, Coefficient; CI, confidence interval; Q1, quintile 1; Ref, reference category.

## Table F: Full model results from Table 3 Model 2 in main manuscript

|  | OR/Coef. | 95% CI | p value |
| --- | --- | --- | --- |
| *Smoker* |  |  |  |
| *Stable non-deprived* | *Ref* | *Ref* | *Ref* |
| *Rising deprivation* | 1.093 | 0.531 to 2.248 | 0.810 |
| *Declining deprivation* | 1.274 | 0.859 to 1.887 | 0.229 |
| *Stable Deprived* | 1.245 | 0.827 to 1.876 | 0.294 |
| Age | 1.319 | 1.028 to 1.690 | 0.029 |
| Male | 0.849 | 0.701 to 1.027 | 0.093 |
| Social class | |  |  |
| *I* | *Ref* | *Ref* | *Ref* |
| *II* | 1.565 | 1.171 to 2.094 | 0.002 |
| *III (non-manual)* | 1.480 | 1.036 to 2.117 | 0.031 |
| *III (manual)* | 1.732 | 1.137 to 2.641 | 0.011 |
| *IV* | 1.876 | 1.093 to 3.222 | 0.022 |
| Parental education | |  |  |
| *Degree* | *Ref* | *Ref* | *Ref* |
| *A level* | 0.889 | 0.691 to 1.141 | 0.355 |
| *O level* | 1.091 | 0.811 to 1.467 | 0.563 |
| *CSE/vocational* | 1.313 | 0.901 to 1.910 | 0.157 |
| Maternal age | 0.994 | 0.969 to 1.018 | 0.605 |
| Parental marriage status | | |  |
| *Married* | *Ref* | *Ref* | *Ref* |
| *Partnered* | 1.206 | 0.886 to 1.640 | 0.234 |
| *Single* | 1.540 | 1.034 to 2.296 | 0.034 |
| Constant | 0.223 | 0.101 to 0.491 | <0.001 |
|  |  |  |  |
| *Weekly cigarettes* | |  |  |
| *Stable non-deprived* | *Ref* | *Ref* | *Ref* |
| *Rising deprivation* | -0.121 | -0.373 to 0.131 | 0.348 |
| *Declining deprivation* | 0.155 | -0.132 to 0.442 | 0.290 |
| *Stable Deprived* | 0.094 | -0.101 to 0.290 | 0.343 |
| Age | -0.072 | -0.266 to 0.122 | 0.468 |
| Male | 0.161 | 0.015 to 0.307 | 0.031 |
| Social class | |  |  |
| *I* | *Ref* | *Ref* | *Ref* |
| *II* | 0.180 | -0.103 to 0.464 | 0.212 |
| *III (non-manual)* | 0.266 | -0.061 to 0.593 | 0.111 |
| *III (manual)* | 0.281 | -0.059 to 0.621 | 0.105 |
| *IV* | 0.290 | -0.121 to 0.701 | 0.167 |
| Parental education | |  |  |
| *Degree* | *Ref* | *Ref* | *Ref* |
| *A level* | 0.111 | -0.118 to 0.341 | 0.342 |
| *O level* | 0.327 | 0.085 to 0.568 | 0.008 |
| *CSE/vocational* | 0.259 | -0.031 to 0.550 | 0.080 |
| Maternal age | -0.020 | -0.037 to -0.004 | 0.017 |
| Parental marriage status | | |  |
| *Married* | *Ref* | *Ref* | *Ref* |
| *Partnered* | 0.248 | 0.047 to 0.449 | 0.016 |
| *Single* | 0.306 | -0.003 to 0.616 | 0.052 |
| Constant | 3.861 | 3.269 to 4.453 | <0.001 |
|  |  |  |  |
| /lnalpha | -0.188 | -0.318 to -0.057 | 0.005 |
| alpha | 0.829 | 0.728 to 0.944 | <0.001 |

OR, Odds Ratio; Coef, Coefficient; CI, confidence interval; Q1, quintile 1; Ref, reference category.

## Table G: Full model results from Table 3 Model 3 in main manuscript

|  | OR/Coef. | 95% CI | p value |
| --- | --- | --- | --- |
| Smoker |  |  |  |
| Stable non-deprived | *Ref* | *Ref* | *Ref* |
| Rising deprivation | 0.945 | 0.463 to 1.923 | 0.875 |
| Declining deprivation | 1.073 | 0.727 to 1.584 | 0.723 |
| Stable deprived | 1.027 | 0.667 to 1.582 | 0.902 |
| Age | 1.331 | 1.030 to 1.719 | 0.029 |
| Male | 0.836 | 0.690 to 1.014 | 0.069 |
| Social class | |  |  |
| *I* | *Ref* | *Ref* | *Ref* |
| *II* | 1.459 | 1.087 to 1.960 | 0.012 |
| *III (non-manual)* | 1.390 | 0.969 to 1.996 | 0.074 |
| *III (manual)* | 1.484 | 0.969 to 2.277 | 0.070 |
| *IV* | 1.667 | 0.968 to 2.872 | 0.065 |
| Parental education | |  |  |
| *Degree* | *Ref* | *Ref* | *Ref* |
| *A level* | 0.835 | 0.647 to 1.078 | 0.166 |
| *O level* | 1.006 | 0.745 to 1.359 | 0.966 |
| *CSE/vocational* | 1.104 | 0.751 to 1.624 | 0.616 |
| Maternal age | 1.003 | 0.979 to 1.028 | 0.794 |
| Parental marriage status | | |  |
| *Married* | *Ref* | *Ref* | *Ref* |
| *Partnered* | 0.943 | 0.683 to 1.302 | 0.722 |
| *Single* | 1.204 | 0.789 to 1.837 | 0.389 |
| Parents who smoke | |  |  |
| *Neither* | *Ref* | *Ref* | *Ref* |
| *One parent* | 1.735 | 1.376 to 2.188 | <0.001 |
| *Both parents* | 3.307 | 2.565 to 4.267 | <0.001 |
| Constant | 0.143 | 0.065 to 0.317 | <0.001 |
|  |  |  |  |
| Weekly cigarettes | |  |  |
| Stable non-deprived | *Ref* | *Ref* | *Ref* |
| Rising deprivation | -0.171 | -0.412 to 0.070 | 0.164 |
| Declining deprivation | 0.115 | -0.184 to 0.414 | 0.450 |
| Stable deprived | 0.082 | -0.114 to 0.279 | 0.412 |
| Age | -0.047 | -0.250 to 0.156 | 0.650 |
| Male | 0.174 | 0.028 to 0.320 | 0.019 |
| Social class | |  |  |
| *I* | *Ref* | *Ref* | *Ref* |
| *II* | 0.159 | -0.115 to 0.433 | 0.256 |
| *III (non-manual)* | 0.230 | -0.096 to 0.556 | 0.167 |
| *III (manual)* | 0.228 | -0.111 to 0.567 | 0.188 |
| *IV* | 0.232 | -0.173 to 0.636 | 0.261 |
| Parental education | |  |  |
| *Degree* | *Ref* | *Ref* | *Ref* |
| *A level* | 0.122 | -0.098 to 0.343 | 0.277 |
| *O level* | 0.325 | 0.087 to 0.563 | 0.007 |
| *CSE/vocational* | 0.255 | -0.035 to 0.546 | 0.085 |
| Maternal age | -0.017 | -0.034 to -0.001 | 0.036 |
| Parental marriage status | | |  |
| *Married* | *Ref* | *Ref* | *Ref* |
| *Partnered* | 0.221 | 0.015 to 0.426 | 0.035 |
| *Single* | 0.301 | -0.016 to 0.618 | 0.063 |
| Parents who smoke | |  |  |
| *Neither* | *Ref* | *Ref* | *Ref* |
| *One parent* | 0.163 | -0.020 to 0.345 | 0.081 |
| *Both parents* | 0.196 | -0.001 to 0.394 | 0.051 |
| Constant | 3.712 | 3.115 to 4.308 | <0.001 |
|  |  |  |  |
| /lnalpha | -0.196 | -0.327 to -0.066 | 0.003 |
| alpha | 0.822 | 0.721 to 0.936 | <0.001 |

OR, Odds Ratio; Coef, Coefficient; CI, confidence interval; Q1, quintile 1; Ref, reference category.

## Table H: Full model results for Figure 2 in main manuscript

|  | OR/Coef. | 95% CI | p value |
| --- | --- | --- | --- |
| *Smoker* |  |  |  |
| *Stable non-deprived* | *Ref* | *Ref* | *Ref* |
| *Rising deprivation* | 1.904 | 7.791 to 0.465 | 0.370 |
| *Declining deprivation* | 1.430 | 2.835 to 0.722 | 0.305 |
| *Stable deprived* | 1.438 | 2.971 to 0.696 | 0.327 |
| Composite SEP | 0.968 | 0.987 to 0.949 | 0.001 |
| *Composite SEP*stable non-deprived* | *Ref* | *Ref* | *Ref* |
| *Composite SEP*rising deprivation* | 0.943 | 1.115 to 0.797 | 0.492 |
| *Composite SEP*declining deprivation* | 0.998 | 1.069 to 0.931 | 0.961 |
| *Composite SEP*stable deprived* | 0.991 | 1.076 to 0.913 | 0.835 |
| Age | 1.326 | 1.702 to 1.033 | 0.027 |
| Male | 0.845 | 1.020 to 0.698 | 0.080 |
| Constant | 0.400 | 0.515 to 0.311 | <0.001 |
|  |  |  |  |
| *Weekly cigarettes* | |  |  |
| *Stable non-deprived* | *Ref* | *Ref* | *Ref* |
| *Rising deprivation* | -0.021 | -0.368 to 0.326 | 0.906 |
| *Declining deprivation* | -0.113 | -0.523 to 0.297 | 0.589 |
| *Stable deprived* | 0.076 | -0.197 to 0.349 | 0.585 |
| Composite SEP | -0.044 | -0.059 to -0.028 | <0.001 |
| *Composite SEP*stable non-deprived* | *Ref* | *Ref* | *Ref* |
| *Composite SEP*rising deprivation* | -0.009 | -0.038 to 0.020 | 0.542 |
| *Composite SEP*declining deprivation* | 0.041 | -0.009 to 0.092 | 0.110 |
| *Composite SEP*stable deprived* | 0.006 | -0.023 to 0.034 | 0.698 |
| Age | -0.015 | -0.209 to 0.178 | 0.878 |
| Male | 0.135 | -0.009 to 0.280 | 0.067 |
| Constant | 4.136 | 3.959 to 4.312 | <0.001 |
|  |  |  |  |
| /lnalpha | 1.182 | 1.347 to 1.037 | 0.012 |
| alpha | 0.429 | 0.476 to 0.381 | <0.001 |

OR, Odds Ratio; Coef, Coefficient; CI, confidence interval; Q1, quintile 1; Ref, reference category; SEP, socioeconomic position.

## Table I: Full model results from Table 5 Model 1 in main manuscript

|  | OR/Coef. | 95% CI | p value |
| --- | --- | --- | --- |
| *Smoker* |  |  |  |
| *Stable non-deprived* | *Ref* | *Ref* | *Ref* |
| *Rising deprivation* | 1.105 | 0.530 to 2.303 | 0.789 |
| *Declining deprivation* | 1.359 | 0.929 to 1.988 | 0.114 |
| *Stable deprived* | 1.571 | 1.053 to 2.347 | 0.027 |
| Age | 1.318 | 1.028 to 1.690 | 0.029 |
| Male | 0.840 | 0.695 to 1.017 | 0.074 |
| Household move | 1.121 | 1.034 to 1.214 | 0.006 |
| Constant | 0.247 | 0.209 to 0.293 | <0.001 |
|  |  |  |  |
| *Weekly cigarettes* |  |  |  |
| *Stable non-deprived* | *Ref* | *Ref* | *Ref* |
| *Rising deprivation* | 0.075 | -0.198 to 0.348 | 0.590 |
| *Declining deprivation* | 0.290 | 0.029 to 0.551 | 0.029 |
| *Stable deprived* | 0.284 | 0.096 to 0.472 | 0.003 |
| Age | -0.060 | -0.254 to 0.134 | 0.546 |
| Male | 0.097 | -0.048 to 0.242 | 0.189 |
| Household move | 0.013 | -0.036 to 0.063 | 0.595 |
| Constant | 3.688 | 3.567 to 3.808 | <0.001 |
|  |  |  |  |
| /lnalpha | -0.109 | -0.239 to 0.021 | 0.100 |
| alpha | 0.897 | 0.787 to 1.021 | <0.001 |

OR, Odds Ratio; Coef, Coefficient; CI, confidence interval; Q1, quintile 1; Ref, reference category.

## Table J: Full model results from Table 5 Model 2 in main manuscript

|  | OR/Coef. | 95% CI | p value |
| --- | --- | --- | --- |
| *Smoker* |  |  |  |
| *Stable non-deprived* | *Ref* | *Ref* | *Ref* |
| *Rising deprivation* | 0.955 | 0.458 to 1.990 | 0.902 |
| *Declining deprivation* | 1.169 | 0.787 to 1.735 | 0.438 |
| *Stable Deprived* | 1.283 | 0.849 to 1.937 | 0.237 |
| Age | 1.294 | 1.009 to 1.660 | 0.043 |
| Male | 0.855 | 0.705 to 1.036 | 0.109 |
| Social class |  |  |  |
| *I* | *Ref* | *Ref* | *Ref* |
| *II* | 1.573 | 1.175 to 2.106 | 0.002 |
| *III (non-manual)* | 1.502 | 1.049 to 2.151 | 0.026 |
| *III (manual)* | 1.770 | 1.158 to 2.702 | 0.008 |
| *IV* | 1.923 | 1.119 to 3.304 | 0.018 |
| Parental education |  |  |  |
| *Degree* | *Ref* | *Ref* | *Ref* |
| *A level* | 0.902 | 0.702 to 1.161 | 0.423 |
| *O level* | 1.106 | 0.823 to 1.487 | 0.504 |
| *CSE/vocational* | 1.331 | 0.915 to 1.939 | 0.135 |
| Maternal age | 1.001 | 0.975 to 1.026 | 0.948 |
| Parental marriage status |  |  |  |
| *Married* | *Ref* | *Ref* | *Ref* |
| *Partnered* | 1.157 | 0.847 to 1.579 | 0.360 |
| *Single* | 1.461 | 0.981 to 2.177 | 0.062 |
| Household moves | 1.104 | 1.013 to 1.203 | 0.024 |
| Constant | 0.160 | 0.068 to 0.380 | <0.001 |
|  |  |  |  |
| *Weekly cigarettes* |  |  |  |
| *Stable non-deprived* | *Ref* | *Ref* | *Ref* |
| *Rising deprivation* | -0.111 | -0.390 to 0.167 | 0.433 |
| *Declining deprivation* | 0.161 | -0.142 to 0.464 | 0.299 |
| *Stable Deprived* | 0.094 | -0.101 to 0.289 | 0.344 |
| Age | -0.072 | -0.266 to 0.122 | 0.469 |
| Male | 0.159 | 0.012 to 0.306 | 0.034 |
| Social class |  |  |  |
| *I* | *Ref* | *Ref* | *Ref* |
| *II* | 0.182 | -0.104 to 0.467 | 0.212 |
| *III (non-manual)* | 0.266 | -0.061 to 0.592 | 0.111 |
| *III (manual)* | 0.281 | -0.059 to 0.622 | 0.105 |
| *IV* | 0.288 | -0.122 to 0.698 | 0.168 |
| Parental education |  |  |  |
| *Degree* | *Ref* | *Ref* | *Ref* |
| *A level* | 0.110 | -0.120 to 0.340 | 0.349 |
| *O level* | 0.326 | 0.083 to 0.568 | 0.009 |
| *CSE/vocational* | 0.259 | -0.031 to 0.548 | 0.080 |
| Maternal age | -0.020 | -0.037 to -0.003 | 0.018 |
| Parental marriage status |  |  |  |
| *Married* | *Ref* | *Ref* | *Ref* |
| *Partnered* | 0.247 | 0.046 to 0.449 | 0.016 |
| *Single* | 0.308 | -0.001 to 0.618 | 0.051 |
| Household moves | -0.005 | -0.056 to 0.046 | 0.853 |
| Constant | 3.877 | 3.265 to 4.488 | <0.001 |
|  |  |  |  |
| /lnalpha | -0.188 | -0.318 to -0.057 | 0.005 |
| alpha | 0.829 | 0.727 to 0.944 | <0.001 |

OR, Odds Ratio; Coef, Coefficient; CI, confidence interval; Q1, quintile 1; Ref, reference category.

## Table K: Full model results from Table 5 Model 3 in main manuscript

|  | OR/Coef. | 95% CI | p value |
| --- | --- | --- | --- |
| *Smoker* |  |  |  |
| Stable non-deprived | *Ref* | *Ref* | *Ref* |
| Rising deprivation | 0.885 | 0.427 to 1.837 | 0.743 |
| Declining deprivation | 1.031 | 0.697 to 1.528 | 0.876 |
| Stable Deprived | 1.041 | 0.674 to 1.610 | 0.856 |
| Age | 1.317 | 1.017 to 1.702 | 0.036 |
| Male | 0.837 | 0.689 to 1.016 | 0.072 |
| Social class |  |  |  |
| *I* | *Ref* | *Ref* | *Ref* |
| *II* | 1.467 | 1.091 to 1.970 | 0.011 |
| *III (non-manual)* | 1.404 | 0.976 to 2.018 | 0.067 |
| *III (manual)* | 1.507 | 0.981 to 2.316 | 0.061 |
| *IV* | 1.690 | 0.98 to 2.915 | 0.059 |
| Parental education |  |  |  |
| *Degree* | *Ref* | *Ref* | *Ref* |
| *A level* | 0.843 | 0.653 to 1.088 | 0.19 |
| *O level* | 1.018 | 0.754 to 1.374 | 0.909 |
| *CSE/vocational* | 1.119 | 0.760 to 1.645 | 0.571 |
| Maternal age | 1.007 | 0.982 to 1.033 | 0.588 |
| Parental marriage status |  |  |  |
| *Married* | *Ref* | *Ref* | *Ref* |
| *Partnered* | 0.928 | 0.672 to 1.283 | 0.650 |
| *Single* | 1.174 | 0.768 to 1.793 | 0.461 |
| Parents who smoke |  |  |  |
| *Neither* | *Ref* | *Ref* | *Ref* |
| *One parent* | 1.725 | 1.366 to 2.177 | <0.001 |
| *Both parents* | 3.258 | 2.517 to 4.221 | <0.001 |
| Household moves | 1.047 | 0.961 to 1.141 | 0.299 |
| Constant | 0.121 | 0.051 to 0.289 | <0.001 |
|  |  |  |  |
| *Weekly cigarettes* |  |  |  |
| Stable non-deprived | *Ref* | *Ref* | *Ref* |
| Rising deprivation | -0.139 | -0.410 to 0.132 | 0.315 |
| Declining deprivation | 0.134 | -0.174 to 0.443 | 0.393 |
| Stable Deprived | 0.080 | -0.116 to 0.277 | 0.423 |
| Age | -0.045 | -0.247 to 0.157 | 0.664 |
| Male | 0.169 | 0.023 to 0.316 | 0.024 |
| Social class |  |  |  |
| *I* | *Ref* | *Ref* | *Ref* |
| *II* | 0.162 | -0.114 to 0.438 | 0.249 |
| *III (non-manual)* | 0.228 | -0.098 to 0.553 | 0.170 |
| *III (manual)* | 0.225 | -0.113 to 0.563 | 0.193 |
| *IV* | 0.225 | -0.178 to 0.629 | 0.274 |
| Parental education |  |  |  |
| *Degree* | *Ref* | *Ref* | *Ref* |
| *A level* | 0.118 | -0.103 to 0.338 | 0.295 |
| *O level* | 0.322 | 0.083 to 0.560 | 0.008 |
| *CSE/vocational* | 0.251 | -0.037 to 0.540 | 0.088 |
| Maternal age | -0.019 | -0.035 to -0.002 | 0.029 |
| Parental marriage status |  |  |  |
| *Married* | *Ref* | *Ref* | *Ref* |
| *Partnered* | 0.217 | 0.012 to 0.423 | 0.038 |
| *Single* | 0.308 | -0.009 to 0.625 | 0.057 |
| Parents who smoke |  |  |  |
| *Neither* | *Ref* | *Ref* | *Ref* |
| *One parent* | 0.169 | -0.015 to 0.352 | 0.072 |
| *Both parents* | 0.208 | 0.004 to 0.412 | 0.045 |
| Household moves | -0.018 | -0.071 to 0.035 | 0.509 |
| Constant | 3.764 | 3.154 to 4.373 | <0.001 |
|  |  |  |  |
| /lnalpha | -0.197 | -0.328 to -0.066 | 0.003 |
| alpha | 0.821 | 0.720 to 0.936 | <0.001 |

OR, Odds Ratio; Coef, Coefficient; CI, confidence interval; Q1, quintile 1; Ref, reference category.
